# TECH preserves global cognition of older adults with MCI compared with a control group: a randomized controlled trial

**DOI:** 10.1007/s40520-023-02659-6

**Published:** 2024-01-20

**Authors:** Noa Givon Schaham, Zvi Buckman, Debbie Rand

**Affiliations:** 1 Department of Occupational Therapy, Faculty of Medicine, Steyer School of Health Professions, Tel Aviv University, Tel Aviv, Israel; 2 Maccabi Healthcare Services, Tel Aviv-Yafo, Israel

**Keywords:** Technology, Tablet, iPad, Cognitive training

## Abstract

**Background:**

Cognitive training using touchscreen tablet casual game applications (apps) has potential to be an effective treatment method for people with mild cognitive impairment (MCI).

**Aims:**

This study aimed to establish the effectiveness of ‘Tablet Enhancement of Cognition and Health’ (TECH), a novel cognitive intervention for improving/preserving cognition in older adults with MCI.

**Methods:**

A single-blind randomized controlled trial with assessments pre-, post-, and at 6-month follow-up was conducted. TECH entailed 5 weeks of daily self-training utilizing tablet apps, facilitated by weekly group sessions. Global cognition was assessed by the Montreal Cognitive Assessment (MoCA), and specific cognitive components were assessed using WebNeuro computerized battery. Short Form Health Survey (SF-12) assessed health-related quality of life (HRQoL). Intention-to-treat analysis was conducted and the %change was calculated between pre–post and between pre–follow-up. Cohen’s d effect size was also calculated.

**Results:**

Sixty-one participants aged 65–89 years were randomly allocated to TECH (*N* = 31, 14 women) or to standard care (*N* = 30, 14 women). Pre–post and pre–follow-up MoCA %change scores were significantly higher in TECH than control (*U* = 329.5, *p* < .05; *U* = 294.5, *p* < .05) with intermediate effect size values (Cohen’s *d* = .52, Cohen’s *d* = .66). Forty percent of TECH participants versus 6.5% of control participants achieved a minimal clinical important difference in MoCA. Pre–post between-group differences for specific cognitive components were not found and HRQoL did not change.

**Discussion and conclusions:**

TECH encouraged daily self-training and showed to preserve global cognition of older adults with MCI. The implementation of TECH is recommended for older adults with MCI, who are at risk for further cognitive decline.

## Introduction

Older adults with mild cognitive impairment (MCI) are at high risk of progressing to dementia within 5–10 years [1]. They experience subtle cognitive deficits in the areas of memory and executive functions (such as planning, problem-solving, and multitasking). Despite these deficits, older adults with MCI are usually independent in their daily living; however, as the cognitive impairment progresses, a gradual decline in daily functioning might occur, especially in complex tasks, such as driving and financial management [2, 3].

Identifying MCI is important for treatment, since providing intervention at this stage can decrease the risk of developing dementia, or at least slow down the cognitive and functional decline [4, 5]. Since pharmacological treatments are currently not available to cure or modify cognitive decline or dementia, other methods such as cognitive training programs to delay the onset and modify the progression of cognitive deterioration are needed [6].

Cognitive enhancement programs for older adults with MCI have recently been offered via technology such as computer software and video games. These programs are enjoyable and motivate older adults; therefore, they have the potential to preserve and enhance cognition [7, 8]. Such training programs have been shown to be effective in improving cognition in people with MCI; some interventions show moderate to large effect sizes [9–11]. However, most studies have used software developed to train specific cognitive components (such as sustained attention, working memory, memory recall, and problem-solving), rather than to enhance global cognition. Global cognition, required for independent and efficient daily functioning, includes an interaction between different cognitive components. Touchscreen tablets, which are now popular devices, might be a good solution for easier use of technology instead of desktop computers. Using touchscreen tablets can also encourage individuals to learn a new cognitive skill in addition to practicing different casual game tablet applications (apps), which might be enjoyable and suitable to use for cognitive training.

Touchscreen tablets have been used by older adults for different purposes in various clinical settings [12], including cognitive training. The effectiveness of cognitive training using tablets was assessed in two randomized controlled trials, resulting in improvements in processing speed [13, 14] and episodic memory [13] in healthy older adults. Other studies included small samples of individuals with dementia; they reported their satisfaction, following a tablet-based leisure program [15] and had a limited improvement in memory and thinking following a tablet-based cognitive training program [16]. Novel programs, such as TECH (Tablet Enhancement of Cognition and Health), aimed to prevent cognitive deterioration in older adults with MCI, are recommended.

The TECH intervention includes daily self-training using tablet casual game apps, facilitated by weekly group sessions. TECH aims to improve global cognition as well as different cognitive components such as memory and executive functions. By playing stimulating casual game apps, TECH provides a leisure activity with high cognitive intellectual stimulation [17], which incorporates new learning that has the potential to improve cognitive function in older adults [18]. The development of TECH has been described elsewhere and the feasibility of using TECH for older adults with MCI was demonstrated [19]. The current study, which included the same older adult participants, aims to determine the effectiveness of the TECH intervention, compared to standard care, for improving global cognition, specific cognitive components, and health-related quality of life (HRQoL) in older adults with MCI. We hypothesized that TECH would maintain or even improve global cognition and specific cognitive components in older adults with MCI, compared with the control group. We also hypothesized that these improvements would have a positive impact on their HRQoL.

## Methods

### Study design

This is a single-blind randomized controlled trial (clinical trial number NCT02955277) with assessments administered pre- and post- the 5-week intervention, and at 6-month follow-up, by assessors blind to group allocation.

### Population

Community-dwelling older adults (> 65 years) were recruited between May 2017 to November 2019 and the follow-up assessments lasted until May 2020, once data collection was complete. The potential participants were recruited from two community geriatric clinics due to their complaints of memory problems, and were referred to the study by their family or geriatric physician. Inclusion criteria were as follows: (a) a diagnosis of MCI, as determined by a score of 19–25 points on the Montreal Cognitive Assessment (MoCA), a valid and reliable tool [20], subjective memory complaints supported by a family member, and independence in basic and instrumental activities of daily living (BADL, IADL), (b) normal or corrected vision and hearing, (c) written and spoken fluency of the language (Hebrew), and (d) ability to use a touchscreen tablet after an initial demonstration. Individuals were excluded if they experienced severe depressive symptoms [Geriatric Depression Scale (GDS) [21] ≥ 10 points], and if they were diagnosed with dementia, or other neurological or psychiatric conditions. This study was approved by the Helsinki Committee of the Healthcare Services (#2016009) and University Ethics Committee and all participants signed informed consent forms before participating in the study.

### Tools

*Outcome measures*: The primary outcome measure used was the MoCA score [20] for assessing global cognition, which was also used to screen for eligibility. Parallel forms of the MoCA were used to avoid a learning effect between the assessments. The WebNeuro assessment [22], a neuropsychological computerized battery, was used for assessing specific cognitive components including: sustained attention (Continuous Performance Task), controlled attention (Verbal Interference Task), flexibility (Switching of Attention Task), inhibition (Go–NoGo Task), working memory (Digit Span Task), memory recall (Memory Recall Task), and problem-solving (Maze Task). Z-scores, with a normative average of 0, and a standard deviation of 1 were calculated for each task. Higher scores indicate better performance. The 12-Item Short Form Health Survey (SF-12) [23], used for measuring the health-related quality of life, was considered a secondary outcome measure. Physical and mental composite summary scores [24] were calculated; higher scores indicated better health measures [23]. All outcome measures are reliable and valid for use with older adults as well as for older adults with MCI [20, 22, 23]. In addition, previous technology experience (e.g., computer, smartphone, and tablet) as well as demographic information was collected.

## The intervention

The TECH intervention (study group) included daily self-training facilitated by weekly group sessions. Participants received iPads to take home and were requested to play casual game apps, which simultaneously provided practicing different cognitive components, at least three to five times a week × 30–60 min, for a total of 15–25 training sessions. Following self-training, participants were asked to log the time they spent performing the self-training, which was recorded. Weekly 1-h sessions led by an occupational therapist (OT) took place in a small group setting (4–6 participants) over a 5-week period. At the beginning of each group session, the OT verified that participants performed the self-training three to five times and encouraged to continue. These sessions focused on teaching tablet operation, allowing participants to explore and practice new apps, and increasing their confidence and independence in using and activating the tablet. Session attendance was monitored.

TECH utilized a variety of apps in terms of complexity and interest to address individual participant’s cognitive level and treatment needs. For the self-training sessions, the OT selected several apps for each participant to play independently at home. Because apps were not specifically developed for cognitive rehabilitation, they required the use and integration of different EF components (and not isolated components), which facilitated practicing different cognitive components, such as working memory, problem-solving, and reasoning. From the options selected, participants could choose what apps to use at home. The development and a detailed description of TECH has been published elsewhere [19].

*Standard care (control group)*: Participants received standard occupational therapy care for MCI including either a) a single consultation in a group setting (4–6 participants) to encourage participants to engage in activities such as solving crossword puzzles or playing board or card games in their leisure time to stimulate cognitive function; or b) participation in a social group (six one-hour sessions of 4–6 participants) of playing puzzle board games, with no recommendation to perform self-training at home.

### Procedure

Participants were approached by phone and provided with information about the study. Those who were willing to participate were invited to the geriatric clinic for the assessment session. After signing an informed consent form, the MoCA, GDS, BADL, and IADL questionnaires were administered to confirm eligibility. If the participants were found eligible, the remaining outcome measures were administered. Then participants were stratified according to low (19–22 points) or high (23–25 points) MoCA score, to assure that groups would be similar in terms of cognitive ability. Then we randomly allocated in blocks (block randomization size 4) using a random number generator app to the TECH or control group using a 1:1 ratio by the principal investigator, who did not take part in the assessments or intervention. Allocation was concealed from the investigators and the enrolled participants. Participants were notified by phone by the study coordinator about their allocation. The assessors, who were blind to group allocation, were OTs trained to assess the study. Participants who were invited to the post- and follow-up assessment sessions were asked not to discuss the intervention with the assessors.

### Data analysis

‘IBM SPSS Statistics 25’ software was used for descriptive statistics to characterize the sample and outcome measures for the three assessments. Intention-to-treat analysis was conducted with the last observation carried forward method [25], which is an acceptable data imputation method [26]. The percentage change was calculated for MoCA, WebNeuro, and SF-12 measurements between pre- and post-intervention and between pre- and follow-up using these formulas [(post–pre)/pre*100%], [(follow-up-pre)/pre*100%]. Since the outcome measures were not normally distributed (including the WebNeuro z-scores percentage change), non-parametric tests were performed. The difference between groups for the percentage change was tested using the Mann–Whitney test. Cohen’s d effect size, which indicates the magnitude of change, was also calculated [27]. First, Cohen’s r effect size values, for non-parametric tests, were calculated by using the following formula [Cohen’s *r* = Z/√N]. Then, the values were converted to Cohen’s d [27]. Cohen’s d effect size were considered small (> 0.1), intermediate (> 0.4), and large (> 0.7) [28]. Effect size indicates clinical meaningfulness, which goes beyond statistical significance, which is also highly dependent on the sample size. In addition, in each group, we calculated the percentage of participants who achieved the minimal clinically important difference (MCID) improvement in global cognition. MCID is defined as the smallest change in scores perceived by the patient as beneficial, which could lead to a change in the patient’s treatment [29]. In this study, the MCID of the MoCA was considered an improvement of at least 1.22 points, as found in stroke rehabilitation [30] (since MCID data for individuals with MCI, to the best of our knowledge, have not yet been established).

The sample size was calculated using ‘G*Power 3.1.9.4’ software, according to a small effect size, based on previous studies assessing the effectiveness of computerized cognitive training for older adults with MCI [11], with 80% power and a significance level of 0.05 [31]. A sample size of 68 participants was calculated. Taking into consideration a 20% dropout rate, 80 participants were required.

## Results

**Fig. 1:**
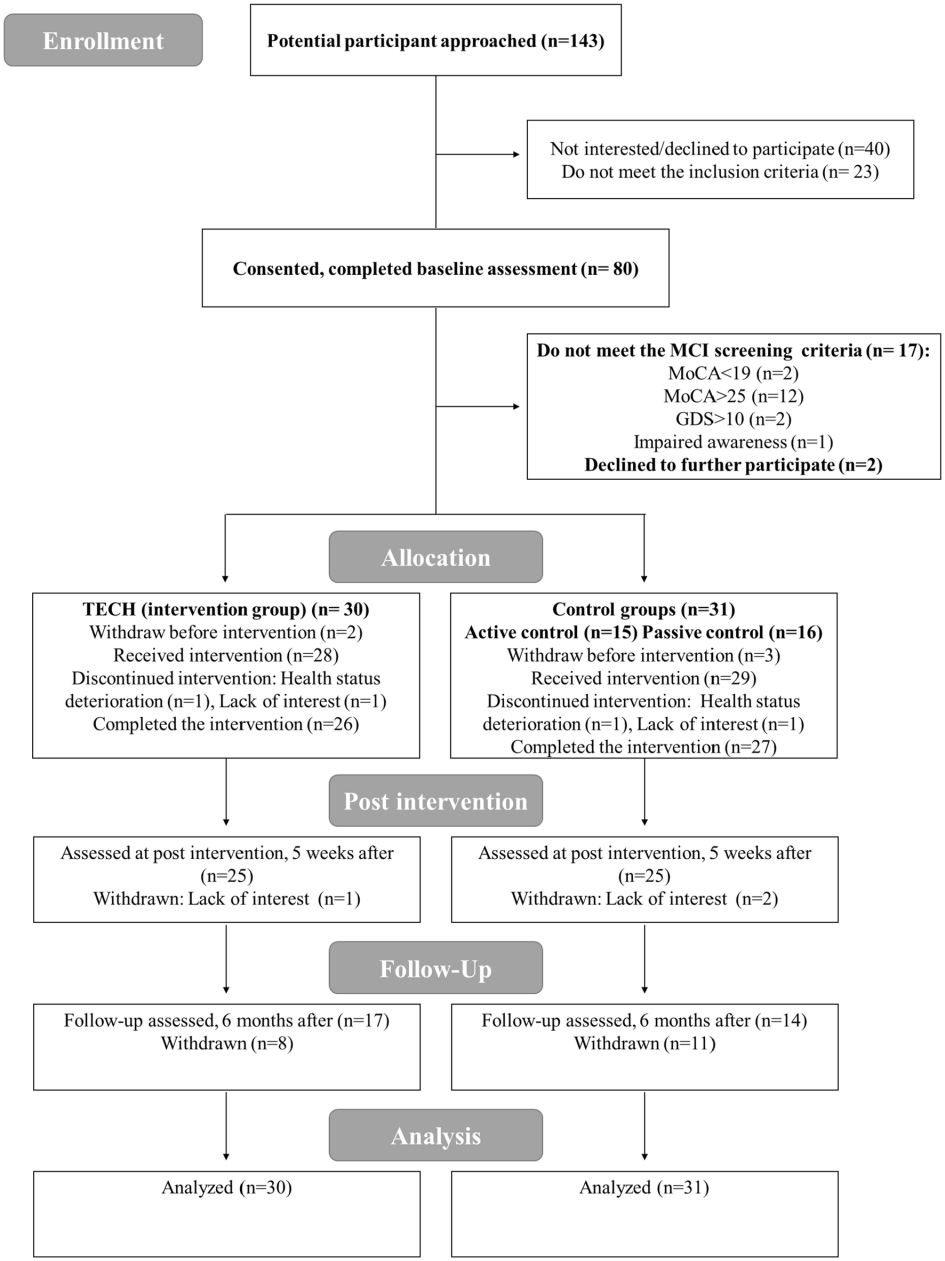
CONSORT flow diagram of the study

Eighty participants were recruited and completed the pre-assessment, 61 participants were found eligible, were randomized, and started the intervention. Fifty of them completed the post-intervention assessment, and 31 underwent the follow-up assessment [see Fig. 1 for a participant flow diagram according to the Consolidated Standards of Reporting Trials (CONSORT) guidelines]. Thirty participants were allocated to the TECH intervention group [14 women and 16 men aged 65–87 (mean age 75.6)] and 31 participants were allocated to the control group [14 women and 17 men aged 65–89 (mean age 75.1)]; 17 received a single consultation and 14 participated in the social group. Since differences in the dependent variables were not found between the two arms of the control group, data from both arms were combined and analyzed as one control group.

All participants had MCI, were independent in BADL and IADL, and were without severe depressive symptoms. Most participants reported using a smartphone and/or a computer on a daily basis prior to the study, and a few reported previous touchscreen tablet experience. No significant differences were found between groups regarding the demographic characteristics (Table 1) or the cognitive status pre-intervention (Table 2). As shown in Table 1, participants from both groups were heterogeneous in terms of cognitive status (MoCA); scores ranged from 19 to 25/30 points.

**Table 1:** Demographic characteristics of the participants in both groups

	TECH (N = 30) Median IQR	CONTROL (N = 31) Median IQR	Mann–Whitney *U* test *U*
Age (years)	74.5 72.0–80.2	74.0 70.0–78.0	439.0
Education (years)	12.0 12.0–14.2	13.0 12.0–16.0	415.5
MoCA (0–30)	23 21–24	23 20–24	422.5

		N (%)	N (%)	χ2
Sex	Female	14 (46.7)	14 (45.2)	.01
	Male	16 (53.3)	17 (54.8)	
Residence	Alone	5 (16.7)	9 (29.0)	1.3
	With family	25 (83.3)	22 (71.0)	
Main occupation	Work	7 (23.3)	5 (16.1)	.50
	Retired	23 (76.7)	26 (83.9)	
Drive	Yes	25 (83.3)	26 (83.9)	.00
Computer use	Yes	24 (80.0)	22 (71.0)	1.1
Smartphone use	Yes	24 (86.7)	29 (93.5)	.81
Tablet use	Yes	8 (26.7)	7 (22.6)	.14

*MoCA* Montreal Cognitive Assessment

*p < .05

**p < .01

**Table 2:** The median, IQR, and min–max scores of the cognitive measures on three assessments of TECH and control groups; and differences between groups pre-intervention

		TECH (N = 30)			CONTROL (N = 31)			Differences between groups pre-intervention*
		Pre-intervention	Post-intervention	Follow-up	Pre-intervention	Post-intervention	Follow-up	
		Median IQR Min–Max	Median IQR Min–Max	Median IQR Min–Max	Median IQR Min–Max	Median IQR Min–Max	Median IQR Min–Max	*p*
MoCA		23.0 21.0–24.0 19.0–25.0	23.0 21.7–25.0 16.0–28.0	23.5 21.7–25.2 14.0–28.0	23.0 20.0–24.0 19.0–25.0	22.0 20.0–24.0 16.0–27.0	22.0 20.0–23.0 15.0–27.0	.53
WebNeuro Computerized Cognitive Battery	Sustained attention	− .5 − 1.0–(− .03) − 2.0–.7	− .4 − .9-.1 − 2.0–.7	− .2 − .8–.05 − 2.0–.8	− .5 − 1.2–(− .1) − 2.2–1.0	− .5 − 1.2–.1 − 2.2–1.0	− .4 − 1.2–.1 − 2.1–1.0	.54
	Controlled attention	− 1.1 − 1.7–(− .6) − 2.2–(− .1)	− 1.3 − 1.7–(− .9) − 2.2–(− .1)	− 1.1 − 1.6–(− .8) − 2.2-(− .1)	− 1.2 − 1.9–(− .8) − 2.2–(− .2)	− 1.3 − 1.7–(− .9) − 2.1–(− .5)	− 1.5 − 1.9–(− .9) − 2.2–(− .5)	.32
	Flexibility	− 1.2 − 1.9–(− .5) − 2.0–.7	− 1.2 − 1.9–(− .7) − 2.0–.7	− 1.3 − 1.9–(− .5) − 2.2–.8	− 1.7 − 1.9–(− 1.3) − 2.1–.2	− 1.7 − 1.9–(− .7) − 2.1–.3	− 1.8 − 1.9–(− .8) − 2.1–.4	.14
	Inhibition	− .3 − .7–.2 − 1.6–.8	− .1 ..5–.3 .1.6–.9	− .05 − .5–.3 − 1.8–.8	− .7 − 1.1–(− .1) − 1.7–.7	− .5 − 1.0–.07 − 1.7–.6	− .5 − 1.0–.1 − 1.9–.7	.12
	Working memory	− 1.4 − 1.8–(− .8) − 2.2–.7	− 1.1 − 1.9–(− .8) − 2.2–1.7	− 1.2 − 1.9–(− .7) − 2.2–1.3	− 1.1 − 1.9–(− .9) − 2.2–.6	− 1.3 − 1.9–(− .9) − 2.2–(− .2)	− 1.2 − 1.8–(− .8) − 2.2–.6	.79
	Memory recall	− .9 − 1.8–.06 − 2.2–.8	− 1.2 − 2.2–(− .5) − 2.2–1.7	− 1.2 − 2.2–(− .7) − 2.2–1.3	− 1.0 − 2.2–(− .4) − 2.2–.8	− 1.4 − 2.2–(− .4) − 2.2–.8	− 1.8 − 2.2–(− .4) − 2.2–1.3	.23
	Problem-solving	.1 − .4–.5 − 2.2–1.5	.3 − .6–.7 − 2.2–1.5	.3 − .4–.6 − 2.2–1.5	.1 − .3–.5 − 2.2–.5	.5 − .1–.9 − 2.2–1.2	.3 − .2–.9 − 2.2–1.2	.73

*Mann–Whitney *U* test

During the TECH intervention, participants attended at least 80% of the six group sessions. Their median (IQR) total self-training time was 23.6 (16.8–29.1) hours, ranging from 5.3–50.1 h over 5-weeks (from 22 participants who filled in the daily log).

**Fig. 2:**
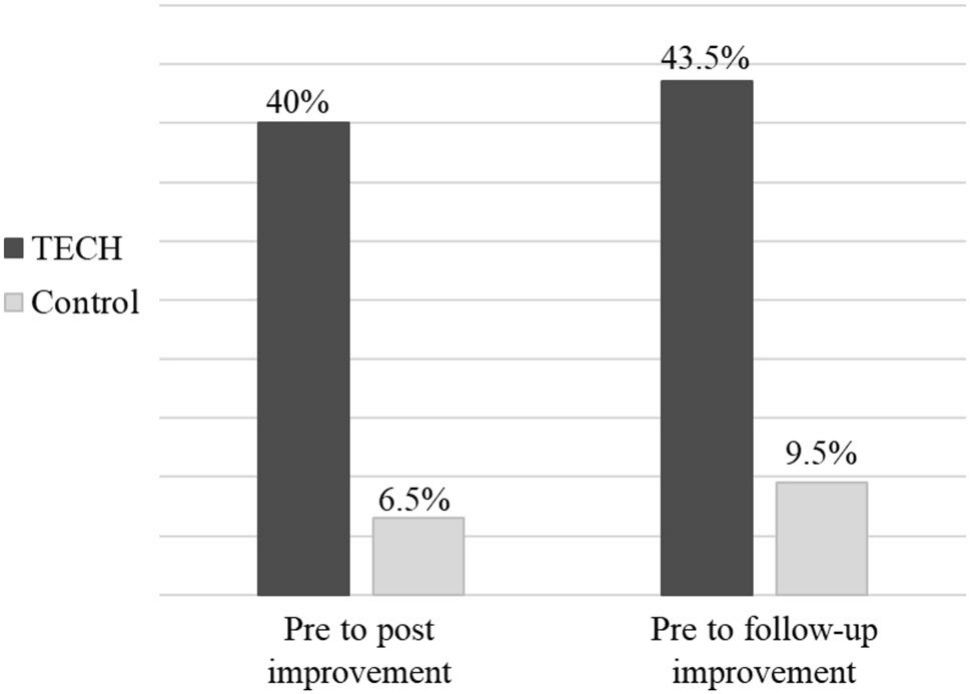
Percentage of participants in the TECH group (black) and control group (gray) who achieved MCID of MoCA scores on post- and follow-up assessments

Table 2 presents the median, IQR and min–max scores of the cognitive measures pre-, post-intervention and on follow-up. Table 3 presents the percent change from pre- to post-intervention and pre-intervention to follow-up. The median (IQR) percentage MoCA change from pre- to post-intervention in the TECH group was 0.0 [(− 4.2)–10.5], compared with 0.0 [(− 5.0)–0.0] in the control group. This difference between groups was statistically significant (*U* = 329.5, *p* < 0.05), with an intermediate effect size (Cohen’s *d* = 0.52). The difference in median (IQR) percentage MoCA change from pre- to follow-up between groups was also significant (*U* = 294.5, *p* < 0.05), with an intermediate effect size (Cohen’s *d* = 0.66). The median (IQR) percentage change was 2.1 [(− 4.2)–12.3] in the TECH group and 0.0 [(− 10.0)–0.0] in the control group. Analysis of the percentage of participants who showed an improvement in MoCA scores revealed that post-intervention 40% of the TECH participants, compared with only 6.5% in the control group, achieved MCID improvement. In addition, 43.5% of the TECH participants showed an improvement from the pre- to the follow-up assessment, but only 9.5% in the control group achieved this improvement (see Fig. 2).

**Table 3:** The median, IQR, and min–max of percentage of change and effect size pre- to post-intervention and pre- to follow-up in each group and differences between groups

		TECH (*N* = 30)		Control (*N* = 31)			Differences between groups pre–post % change*	Differences between groups pre–follow-up % change*	Cohen’s d Pre–post	Cohen’s d Pre–follow-up
		% change Pre–post	% change Pre–follow-up		% change Pre–post	% change Pre–follow-up				
		Median IQR Min–Max	Median IQR Min–Max		Median IQR Min–Max	Median IQR Min–Max	(*p*)	(*p*)		
MoCA		0.0 (− 4.2)–10.5 − 20.8–19.0	2.1 (− 4.2)–12.3 − 30.0–21.7		0.0 (− 5.0)–0.0 − 15.8–17.4	0.0 (− 10.0)–0.0 − 25.0–17.3	.04	.01	.52	.66
WebNeuro computerized Cognitive battery	Sustained attention	− 28.9 (− 90.1)–49.1 − 759.1–221.9	− 26.1 (− 78.9)–0.0 − 318.9–480.4		0.0 (− 77.6)–18.4 − 281.9–169.4	− 6.9 (− 123.3)–3.9 − 386.1–198.3	.92	.65	.02	.12
	Controlled attention	0.0 (− 13.1)–48.59 − 55.4–419.7	0.0 (− 17.1)–41.5 − 66.8–419.7		0.0 (− 26.1)–24.7 − 61.8–584.1	0.0 (− 18.0)–45.9 − 61.7–274.4	.76	.86	.08	.05
	Flexibility	0.0 (− 35.4)–10.1 − 1146.2–462.4	− 0.12 (− 39.9)–7.5 − 1462.5–411.3		− 0.15 (− 18.9)–0.0 − 144.6–39.0	− 0.13 (− 0.65)–0.01 − 188.1–57.9	.24	.95	.30	.01
	Inhibition	− 44.1 (− 130.8)–39.7 − 1153.8–864.8	− 47.8 (− 131.2)–31.1 − 2096.2–163.8		− 1.4 (− 39.6)–0.0 − 113.9–224.6	− 1.5 (− 44.1)–12.8 − 377.3–224.6	.27	.45	.39	.29
	Working memory	− .28 (− 42.9)–11.6 − 259.1–883.1	− 1.8 (− 39.3)–0.0 − 224.7–1162.4		0.0 (− 17.4)–21.6 − 277.1–426.6	0.0 (− 12.6)–16.1 − 79.0–135.4	.45	.17	.21	.38
	r	0.0 (− 57.2)–84.9 − 338.1–740.5	0.0 (− 155.9)–20.6 − 992.8–684.7		0.0 (− 9.7)–12.4 − 256.6–4852.8	0.0 0.0–45.0 − 212.4–4852.8	.98	.19	.00	.33
	Problem-solving	0.0 (− 134.0)–60.4 − 3295.0–610.0	− 3.4 (− 115.9)–55.1 − 4472.6–334.1		0.0 (− 124.4)–59.8 − 625.7–1037.7	0.0 (− 147.3)–133.8 − 639.6–1037.7	.97	.86	.01	.05
SF12—Physical Composite Score		0.0 0.0–0.0 − 31.2–22.67	0.0 0.0–0.0 − 31.2–45.33		0.0 0.0–0.0 − 31.9–45.3	0.0 0.0–0.0 − 18.5–45.3	.06	.85	.36	.04
SF12—Mental Composite Score		0.0 0.0–0.0 − 37.8–53.7	0.0 0.0–0.0 − 41.7–53.7		0.0 0.0–0.0 − 28.3–65.4	0.0 0.0–0.0 − 34.9–65.4	.74	.46	.08	.17

Cohen’s r effect size values: small (0.1), medium (0.3), and large (0.5)^27^

*Mann–Whitney *U* test

The median percentage change in specific cognitive components, as assessed by the WebNeuro cognitive battery, did not show significant between-group differences; however, a small-intermediate effect size was found from pre- to post-intervention for flexibility (Cohen’s *d* = 0.30), inhibition (Cohen’s *d* = 0.39), and working memory (Cohen’s *d* = 0.21), and from pre- to follow-up for sustained attention (Cohen’s *d* = 0.12), inhibition (Cohen’s *d* = 0.29), working memory (Cohen’s *d* = 0.38), and memory recall (Cohen’s *d* = 0.33).

Significant between-group differences were not found for percentage change for the secondary outcome measure. In addition, no significant correlations were found between self-training time to percentage MoCA change from pre- to post-intervention. However, an intermediate effect size was found for the SF-12 Physical Composite Score from pre- to post-intervention (Cohen’s *r* = 0.36) and for the SF-12 Mental Composite Score from pre- to follow-up (Cohen’s *d* = 0.17) (see Table 3).

## Discussion

Older adults with MCI seek interventions such as TECH to treat and further prevent their subjective memory problems (perceived cognitive decline) [32]. This randomized controlled trial demonstrated the effectiveness of TECH for maintaining and improving global cognition, compared with standard care, but with no advantage in terms of specific cognitive components, or quality of life. TECH is an occupational therapy novel cognitive intervention. This intervention taught how to operate touchscreen tablet devices which facilitates the learning of a new cognitive skill and utilize different apps as a cognitive leisure activity. Participants were taught how to use every-day functional apps (such as the camera, news sites, YouTube) and to play puzzle-game apps, which trained their cognitive abilities while participation in cognitive leisure activities which incorporate learning of new cognitive skills has been found to be associated with a reduced risk of developing dementia [33].

TECH motivated the participants to perform daily self-training over the 5-week intervention period. The high self-training time as well as the high adherence, compliance, and satisfaction from TECH, reported earlier [19], support previous studies. These studies reported enjoyment from using tablet apps and a desire for further practicing, showing high levels of motivation for learning tablet usage [8, 10, 34]. Performing cognitive training several times a week, as was achieved with TECH, has been found to reduce the risk of dementia by a further ~ 50% compared to training performed only once-a-week [33]. In addition, TECH provided participation in cognitive leisure activities, which incorporated using a computer platform (touchscreen tablet) and the learning of new cognitive skills. This may have enhanced the cognitive stimulation [35] which has been associated with a reduced risk of developing dementia [33].

The MoCA, a well-known and commonly used tool with older adults with MCI, was used in our study as a tool for screening MCI, in addition to its use as an outcome measure. MoCA assesses different domains of cognition and executive functioning; the total score reflects global cognition. This test is known to be sensitive to changes in MCI [20] and has alternative versions, making it ideal to use for repeated assessments [36, 37]. Since the participants had MCI, their pre-intervention MoCA scores were limited to be from 19 to 25 points. Nevertheless, a significant improvement with an intermediate effect size was found for the participants in the TECH group, and a high percentage of them achieved a MCID. In the control group no significant improvement in MoCA scores was found and a slight deterioration was observed over time. Only a small percentage of the control group participants achieved the MCID. These positive findings are of paramount importance, since maintaining global cognition of individuals with MCI is crucial for maintaining independent living in the community [38] and for preventing deterioration to dementia [32].

The 6-month follow-up assessment showed a similar trend; participants in the TECH group continued to improve in global cognition, whereas those in the control group did not change. These results reinforce the fact that cognitive training using tablet casual games and functional apps may contribute to maintaining the cognitive status, and consequently, maintain independent living over time among older adults with MCI. The participants' desire to continue practicing even after study completion is very encouraging, since it might further help prevent future deterioration.

The TECH intervention included the use of different casual games and functional apps, thus simultaneously stimulating different cognitive components and therefore training global cognition. Thus, the self-training sessions were stimulating and interesting and training time was overall high. Similar improvements in global cognition following computerized cognitive training for people with MCI were reported in two recent meta-analysis studies, with a small-to-moderate pooled effect size of 0.23–0.38, compared with control groups, which exhibited no cognitive change [10, 11].

However, specific cognitive components, as assessed by the Webneuro computerized battery, showed neither statistically significant improvements nor deterioration for either group. This hypothesis was not verified possibly since this cognitive battery was too difficult and not sensitive enough for older adults with MCI [39]. The scores of participants who were unable to complete the task in the allocated time were not registered. However, small-intermediate effect size values were found for the following cognitive components: sustained attention, flexibility, inhibition, working memory, and memory recall. Perhaps these changes would reach significance with larger samples. Similar findings of no significant improvement in specific cognitive components were reported regarding the effectiveness of a tablet-based intervention for a small sample of older adults with normal cognition [12], or an improvement found in only one component (processing speed) [14].

As opposed to our hypothesis, the cognitive training using TECH did not significantly impact the quality of life of the participants; however, a small-intermediate effect size for SF-12 Physical Composite Score and Mental Composite Score were found. It is unclear why an improvement in the quality of life was not found, especially since 40–43% of the TECH participants achieved the MCID in global cognition. It has been suggested that people with MCI, who are aware of their diagnosis, tend to report a lower quality of life than those who are unaware of their diagnosis [40, 41] Possibly, since all of our participants were aware of their diagnosis and reported low satisfaction with their daily life and physical well-being, a change in their quality of life was not seen post-intervention or at follow-up.

Despite the high adherence with the intervention and self-training, there was difficulty in retaining the participants for the 6-month follow-up assessment. Dropouts are a major problem in RCTs [42] especially in populations with MCI, which is a progressive (but not a dangerous) condition, leading to limited commitment, especially 6 months later.

The control group received standard cognitive treatment as is provided by OTs in many geriatric clinics, either as a one-time consultation session with a recommendation to engage in cognitive stimulating activities, or by participating in a weekly social group focused on playing board games. Standard care was not found to be effective in improving or preserving the participants' cognitive status. In the future, TECH could be implemented as the standard care for this population, aiming to preserve their cognitive status and daily independence.

The limitations of the study include the relatively small sample of older adults with MCI. Although we recruited 80 older adults, only 61 of them were eligible, randomized and started the intervention. In addition, for the 6-month follow-up assessment, we had a high percentage of dropouts. To estimate the treatment effect, we therefore used intention-to-treat analysis, which is considered conservative. Interestingly, participants who dropped out from the control group were significant younger (median age 74) than participants who were included at follow-up (median age 77), however, participants who dropped out of TECH were not significantly different in age or MoCA than the participants who were included at follow-up. In addition, the computerized cognitive battery was not sensitive enough to identify changes in specific cognitive domains, possibly due to the limited time to complete the task and since the z-scores were not corrected for age among other factors. Adding a tool to assess functional cognition could have helped us better understand how the cognitive status of our participants impacts their daily functioning. Although we collected the iPads during the follow-up period, we do not know if participants continued to play games. All participants were free to choose to take part in any type of activity during that time. Due to these limitations, these findings should be interpreted with caution.

## Conclusions and implications

TECH encouraged older participants with MCI to perform daily practice of casual game apps and functional apps, therefore providing a stimulating leisure activity. TECH was found to be effective post-intervention and at 6-month follow-up for preserving and improving global cognition (but not specific cognitive components) in older adults with MCI, compared with a control group. Therefore, TECH is recommended, which may help preserve global cognition in older adults with MCI, who are more vulnerable to further cognitive decline.

## Data Availability

Data sets generated during the current study are available from the corresponding author on reasonable request.
